# Clinical Trial Ethical Challenges in Low- and Middle-Income Countries: A Review of Systematic Review

**DOI:** 10.21203/rs.3.rs-8292062/v1

**Published:** 2025-12-12

**Authors:** Kai Hong Ooi, Pei Boon Ooi, Hui Jie Jaclyn Teng, Chia Wei Phan

**Affiliations:** University of Malaya; Sunway University; Sunway University; University of Malaya

**Keywords:** ethics, clinical trial, low-and-middle-income countries, umbrella review, special population

## Abstract

Clinical trials are increasingly being conducted in low- and middle-income countries (LMICs) for many reasons. However, there are many ethical implications of conduction clinical trials in LMICs. Henceforth, we performed an umbrella review to delineate the evidences regarding the ethical considerations of clinical trial in LMICs. This review followed the PRISMA guideline for the search strategy and used AMSTAR 2 for literature appraisal. This study was registered in PROSPERO (CRD42022325204) to promote transparency and prevent unintended duplication. The search retrieved 18,348 records in stage 1 (16,383 after deduplication); 299 articles were screened for eligibility in stage 2, and eight systematic reviews met inclusion criteria and were included in the umbrella review. AMSTAR-2 appraisal rated three reviews as high confidence, one as low confidence, and four as critically low confidence. Our result shows that informed consent was the most reported ethical issue, and other concerns such as inclusion of vulnerable population, responsible conduct of research and underreporting ought not to be overlooked as well. The communities in LMIC, not being fully aware of their rights, and considering they may lack knowledge of the history of frequent exploitation and bigotry, are more likely to have ethical concerns than wealthy nations. To attain health justice, ethical standards now in place must be upheld and implemented.

## 1 Introduction

Clinical trials play a central role in evaluating novel interventions and medical technologies. They are often the fastest way to establish which therapeutic interventions and diagnostic tests are most applicable to the public. As of April 2022, ClinicalTrials.gov lists more than 400,000 clinical studies worldwide [1].

Clinical trials are frequently conducted in low- and middle-income countries (LMICs) because they can be easier and less expensive to run. Development of clinical research infrastructure in LMICs often increases local access to medical care that would otherwise be unavailable [2]. However, clinical research in LMICs raises important ethical issues that must be addressed to avoid perpetuating health disparities and undermining the trust essential for cross-cultural research [3]. When international collaborations adhere to ethical standards such as the CIOMS Guidelines and to scientific best practices, LMICs can benefit from appropriately conducted domestic clinical trials.

Lang and Siribaddana (2012) described how trials conducted in developing countries can contribute to resource and infrastructure development [4]. At the same time, ethical harms often arise from the actions of specific actors (for example, sponsors, investigators, or institutional systems) operating within contexts of structural power imbalance. Situations where inequitable partnerships, weak governance, and asymmetrical power relations enable exploitative conduct; contemporary scholarship therefore emphasises governance, capacity building, and equitable partnership models as remedies [3]. The internationalisation of clinical trials should not be about exploiting low-cost sites; rather, it can expand research to previously under-represented populations and enable communities to share the benefits of new drugs, vaccines, and improved health-management systems [4].

Experience gained from working with industry sponsors can enhance research quality, improve population health, and bring much-needed resources to research institutions [3]. Nevertheless, barriers and ethical risks remain. Major ethical issues reported in the literature include poor-quality informed consent processes, weak scientific and ethical review, suboptimal regulatory systems for new drugs and trials, inadequate safeguards for patients’ rights and compensation for trial-related harms, and limited post-trial access to proven therapies [5]. Therefore, it is critical that healthcare professionals conducting clinical trials understand their ethical responsibilities and remain aware of the laws and policies that govern their work [6].

In LMICs, poverty, disempowerment, inequity, concerns about exploitation, and culturally distinct beliefs can create public scepticism toward clinical trials [7]. Many barriers can be mitigated through careful planning and collaborative engagement among sponsors, local investigators and sites, government agencies, and communities [8]. Balancing participants’ autonomy and well-being with the need to increase access to medical therapies is essential, and strict compliance with current standards helps ensure scientific validity and that research better reflects the needs of LMIC populations.

Although many single-topic or country-level systematic reviews examine specific ethical problems in clinical research (for example, consent quality, post-trial access, or ethics-committee functioning), there is no higher-level synthesis that aggregates findings across systematic reviews to identify cross-cutting themes and methodological/reporting gaps. Ethical topics are often described using heterogeneous terminology (e.g., “consent”, “community engagement”, “post-trial obligations”), which increases the risk that relevant evidence is scattered across separate reviews and difficult for policymakers to interpret collectively. An umbrella review (overview of systematic reviews) is therefore an appropriate design to: (1) synthesise recurring ethical issues across systematic reviews of clinical trials in LMICs, (2) quantify how consistently these themes appear across the review literature, and (3) identify methodological weaknesses and reporting gaps that impede evidence-based policy.

This study aims to investigate which ethical challenges in clinical trials conducted in low- and middle-income countries (LMICs) have been documented in published systematic reviews, and where evidence gaps remain. We used a PICOST framework to define the review scope: Population (clinical trial participants and communities in LMICs); Intervention/exposure (ethics-related practices and procedural aspects of clinical trials); Comparator (not applicable across many themes); Outcomes (ethical considerations raised); Study types (systematic reviews); Timeframe (all records up to 1 April 2022). We present the review workflow using the PRISMA framework. To increase analytic value beyond description, we appraised methodological quality of included reviews using AMSTAR-2 and mapped the themes to a normative ethical framework to generate policy-relevant conclusions. This approach allows stakeholders to see not only which issues recur in the literature, but also how robust the review evidence is and what practical priorities emerge from higher-level synthesis.

## 2 Methods

### Research design & Search strategy

**Figure 1:**
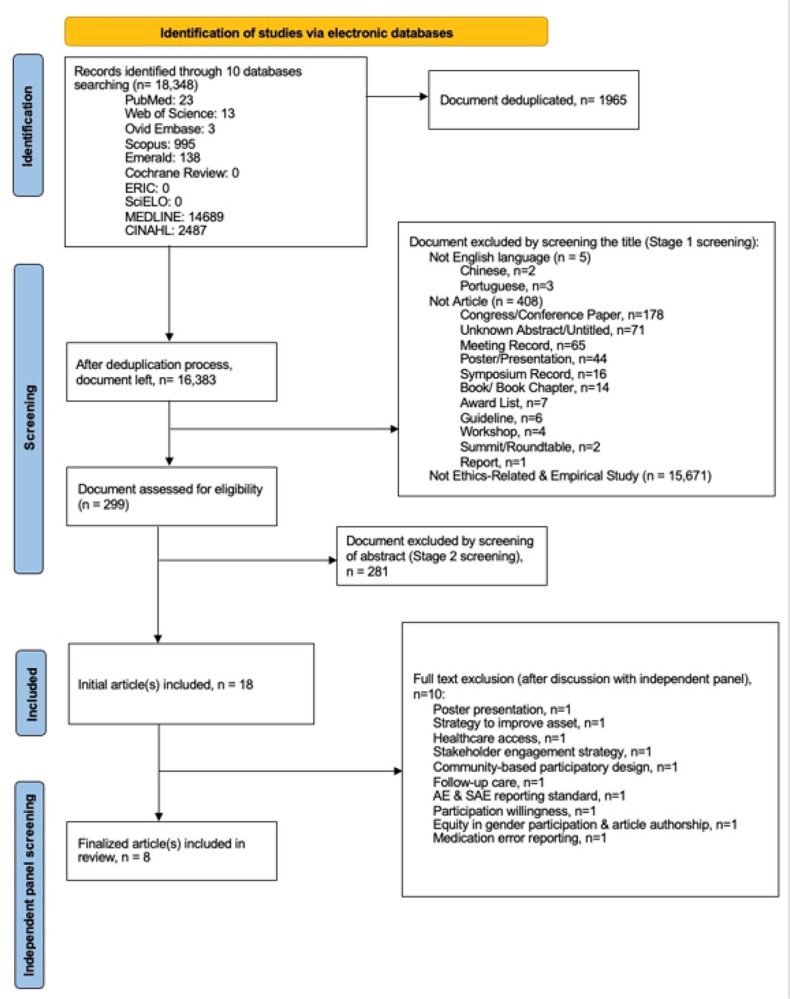
PRISMA workflow adapted for systematic literature review on the ethical consideration in clinical trials among low-and-middle-income countries.

This umbrella review followed the Preferred Reporting Items for Systematic Reviews and Meta-Analyses (PRISMA) guidelines for identification and reporting of included materials (Fig. 1). The review protocol was registered in PROSPERO (CRD42022325204) to reduce the risk of unintended duplication.

We searched ten electronic databases on 1 April 2022: PubMed, Web of Science, Ovid EMBASE, Scopus, Emerald, Cochrane Library, Education Resources Information Center (ERIC), Scientific Electronic Library Online (SciELO), MEDLINE, and CINAHL (Cumulative Index to Nursing and Allied Health Literature). The search used a combination of controlled vocabulary and free-text terms to capture ethics-related systematic reviews of clinical trials in low- and middle-income countries (LMICs). Search terms included “ethic*”, “clinical trial*”, “clinical research”, “low- and middle-income countr*”, and “systematic review*”; synonyms and variant spellings were connected with Boolean operators (AND, OR) and the asterisk (*) was used as a wildcard. Full, refined search strings are provided in Supplementary Table S1. Because ethics-related literature uses heterogeneous terminology and indexing (for example, relevant reviews may discuss “consent”, “community engagement”, or “post-trial obligations” without the label “ethics”), we prioritized sensitivity in the initial search to minimize missed records.

### Deduplication process

Search outputs from each database were exported to Mendeley Desktop (Version 1.19.8). We first ran a primary deduplication within each individual database folder using Mendeley’s “Check for Duplicates” function. Duplicate records were merged within each folder. Duplicates were identified primarily in the Scopus, MEDLINE, and CINAHL folders; no duplicates were found in the other database folders during this stage. After per-database deduplication, all records were compiled into a single master folder in Mendeley and a secondary deduplication step was performed to remove any remaining duplicate records across databases.

### Screening process

Screening proceeded in two stages. In Stage 1 (title screening) we excluded clearly irrelevant records based on the title and our inclusion criteria: (i) systematic reviews addressing clinical research ethics-related issues in low- and lower-middle-income countries (LMICs), and (ii) English language publications only. Stage 2 (abstract screening) involved review of abstracts to assess whether the article met the inclusion criteria; this step was necessary because the initial searches were run across “All Fields” and therefore returned records in which the search terms did not appear in the title.

Records were excluded at Stage 2 for any of the following reasons:

1. Non-English language publication.
2. Publication type other than a systematic review (e.g., book chapter, symposium paper, conference paper, meeting report).
3. Publications that were not systematic reviews (e.g., narrative reviews, commentaries), primary empirical studies (e.g., trial reports, protocols), or papers not related to ethics (e.g., economics, psychology, finance, nutrition).

### Quality appraisal

Articles included in this review were appraised using the AMSTAR 2 (A MeaSurement Tool to Assess systematic Reviews) instrument, in addition to following PRISMA reporting guidelines [31]. We used AMSTAR 2 because umbrella reviews have previously been criticised for variable quality and potential methodological weaknesses [32]. A structured quality checklist (Supplementary Table 3) was therefore applied to enhance interpretation and minimise bias in the process of creating an overview [33].

Two reviewers (O.K.H., first author, and J.T.H.J., third author) independently assessed each included article against the 16 AMSTAR 2 domains [34]. Discrepancies were resolved through discussion and, when necessary, arbitration by the corresponding author until consensus was reached.

## 3 Results

### Screening of data and selection of articles for an umbrella review

Figure 1 delineates the PRISMA workflow adapted from Page et al (2021). The total output obtained was 18,348 documents. After deduplication, 1965 duplicates were removed, and the total compiled document was 16,383. After Stage 1 screening, a total of 16,084 documents were removed. The documents excluded during Stage 1 were 5 non-English articles, 178 conference papers, 71 untitled documents, 65 meeting records, 44 posters, 16 symposium records, 14 book chapters, 7 award lists, 6 guidelines, 4 workshop records, 2 summit documents, 1 unknown report, and lastly a total of 15,671 non-ethics related papers as well as empirical studies. Then, the documents were further filtered (Stage 2 screening) by reading the abstract. A total of 281 articles were removed because they were either not systematic reviews, did not cover LMICs, or did not cover ethical issues.

After full text review and in-depth discussion by all authors, ten articles were excluded. The decision was made due to the nature of the publication, which included a poster presentation (n = 1). The remaining postings were deemed unsuitable for this review, which included: the discussion of strategies to improve assent (n = 1); healthcare access (n = 1); stakeholder engagement strategy (n = 1); community-based participatory research (n = 1); follow-up care (n = 1); adverse event (AE) and severe adverse event (SAE) risk probability reporting (n = 1); willingness of participation (n = 1); equity in gender participation and authorship (n = 1); and, reporting of medication error (n = 1). The excluded articles and reasons for exclusion are described in Supplementary Table S2. They were excluded mainly due to not focusing on ethics concerns in clinical trials and/or not reporting about LMICs.

Table 1 depicts the eight systematic reviews which were included in this review. We further appraised the eight articles using AMSTAR2. While no study met all 16 criteria in AMSTAR 2 upon appraisal (see Supplemental Table S4), some fulfil critical domains. Overall, four articles were determined as critical-low confidence ratings, while three have a high confidence rating. Notably, only one publication has a low confidence rating (Table S3, Supplementary file).

### Low-middle income countries

**Figure 2:**
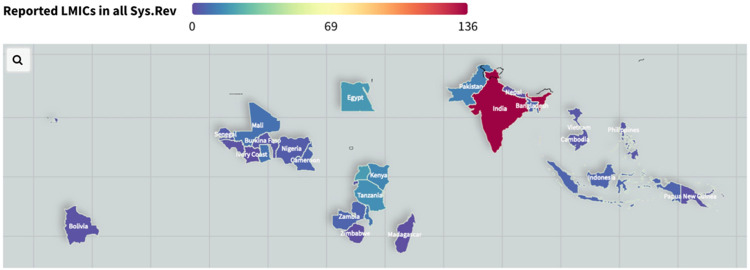
Low-and-middle income countries reported in the eight articles. Colorbar indicate the frequency of countries mentioned in the articles.

A total of 33 countries were reported in the eight articles. The countries (alphabetical order followed by the frequency of reporting, n) are: Bangladesh (n = 6); Benin (n = 3); Bolivia (n = 2); Burkina Faso (n = 3); Cambodia (n = 3); Cameroon (n = 6); Côte d’Ivoire (n = 1); Egypt (n = 18); Gambia (n = 6); Ghana (n = 9); Guinea (n = 2); Guinea-Bissau (n = 2); Haiti (n = 3); India (n = 136); Indonesia (n = 6); Ivory Coast (n = 1); Kenya (n = 14); Madagascar (n = 1); Malawi (n = 10); Mali (n = 9); Nepal (n = 1); Nigeria (n = 4); Pakistan (n = 12); Papua New Guinea (n = 2); Philippines (n = 3); Rwanda (n = 2); Senegal (n = 4); Sub-Saharan Africa (n = 4); Tanzania (n = 16); Uganda (n = 19); Vietnam (n = 3); Zambia (n = 6); and Zimbabwe (n = 1). Figure 2 illustrates the total number of LMICs reported in the eight articles on a map. For instance, India was the most reported country (n = 136) among the systematic reviews (red colored), while the blue-colored countries were reported once or twice respectively. Notably, Uganda was the second most-mentioned LMIC among the eight systematic reviews.

### Target populations and the associated ethical implications

The key populations reported in the eight systematic reviews are pregnant women, people living with HIV, children, minor parents, neonates, indigenous communities, and laypersons. Table 2 summarizes the ethical implications that are related to the population taking part in clinical trials reported in the selected articles. Informed consent was the most mentioned ethical issue.

## 4 Discussion

This umbrella review’s main goal was to comprehensively summarize the systematicreviews on ethical issues related to clinical trials carried out in low- and lower-middle-income countries. The findings are reported in four broad areas which emerged in this review: (1) special and vulnerable populations; (2) informed consent; and (3) responsible conduct of research and underreporting.

### Special and vulnerable populations

Beeler et al. delved into the ethical issues surrounding vaccine trials involving pregnant women. Involvement of pregnant women in clinical trials demands the attention of investigators, particularly during the recruitment phase, to address concerns pertaining to safety, off-label investigational product utilization (in this case, vaccine), and cost and accessibility hurdles [13]. Besides, the ethical issues surrounding the participation of HIV-positive pregnant women (PWLHIV) and their children in clinical trials were thoroughly examined by Raciti et al (2021). The study emphasized the need for a careful balance in managing the risks to both the mother and the fetus, the necessity of obtaining informed consent, the value of paternal involvement, and the significance of ensuring fair access to research opportunities and treatment alternatives[14]. The authors emphasized that pregnant women should be included in trials involving antiretroviral drugs. The controversial dolutegravir drug trial made clear how essential it is to collect timely and pertinent data in order to address concerns for PWLHIV regarding teratogenicity, safety, pharmacokinetics, dose, and effectiveness[15]. The study also highlighted the issue of community stigmatization, which discourages PWLHIV from declaring their health status and taking part in HIV-related research projects[16–17].

Fitzpatrick et al.(2016) concentrated solely on one low-income country (Malawi). The ethical implications of involving marginalized populations, such as indigenous communities or aboriginal peoples, in clinical trials, should not be overlooked. Non-indigenous research teams frequently lack a comprehensive understanding of the multifaceted cultural origins and ideologies of indigenous individuals [18]. Concerns have been raised regarding the potential exclusion of indigenous people from important trials due to perceived exploitation or unequal treatment of indigenous researchers in scholarly publications[19–20]. Consequently, this obstructs the potential for indigenous peoples to actively contribute to mitigating significant health disparities while simultaneously benefiting from research outcomes. Notably, the World Medical Association 2013 guidelines neglect to include indigenous communities as a distinct category warranting special attention, whereas others emphasize the rights of indigenous peoples and stress the imperative of consulting community leaders regarding consent procedures and information dissemination [18, 21–22].

### Informed consent

A noticeable incongruity emerged regarding consent, with most studies noting the attainment of written consent or the waiving of consent under ethical review committee discretion. However, this revelation raises queries about the reporting standards employed in clinical trial publications and the ethical integrity of securing informed consent from prospective participants [13]. These recurrent weaknesses in how consent is reported and obtained also point to system-level failures in ethical oversight. One practical and evidence-supported response is to strengthen Research Ethics Committees (RECs), for instance, competency-based training in protocol appraisal, informed-consent assessment, conflict-of-interest management, and monitoring of post-trial obligations helps RECs identify inadequate consent processes and require corrective actions. Strengthened administrative support (secretariat functions, standardized application forms, and conflict-of-interest registers) and routine REC training have been recommended by WHO and CIOMS as core capacity-building measures to ensure consistent application of ethical standards and improve reporting of ethics procedures in publications. Raciti et al. (2021) found flaws in the informed consent procedure, with no clear advice offered on assuring concerns for PWLHIV, which is consistent with the findings of Beeler et al. (2016). To retain the autonomy and capacity for decision-making of research participants, it is essential to ensure that they have a thorough grasp of the risks and advantages of participation as well as to maintain equilibrium while considering prospective therapies.

In sub-Saharan African studies, there exists an opposing perspective on children’s autonomous consent. De Pretto-Lazarova et al.(2020) discovered difficulties in getting consent from parents who are still minors. The intricacy of the problem was increased by the difficulties of adult proxy decision-making and contextual elements including gender relations and a lack of documentation [23].

The greater consent rates in newborn studies carried out in LMICs as opposed to high-income countries (HICs) were brought to light by Patterson et al. (2021) However, they voiced doubts regarding the efficacy of protections for vulnerable populations and pointed out problems with the informed consent process’s disclosure, understanding, free choice, and authorization steps. The consenting process in LMICs was questioned, including incomplete disclosure, poor understanding, and pressured permission [24].

Similarly, Tam et al. (2015) found that compared to other populations, trial participants in less developed countries showed a worse grasp of their rights to voluntarily participate in studies and to withdraw from them. Furthermore, during a 30-year period, their data showed little progress in participants’ comprehension of possible risks, side effects, and withdrawal freedom [25]. These results highlighted the requirement for improved comprehension and the informed consent procedure.

Participant compensation requires careful calibration: while fair reimbursement is ethical, excessive payments can function as undue inducement and threaten voluntary choice. Another consent-related challenge is **therapeutic misconception**, where participants (especially vulnerable groups) confuse research aims with individualized care and therefore underestimate risks or overestimate benefit. Mitigations include plain-language explanations of the research purpose and potential benefits/risks, comprehension checks during consent, community engagement to set realistic expectations, and active REC review of compensation schemes.

### Responsible conduct of research and underreporting

The surge in clinical trials conducted in LMICs has been found to be accompanied by numerous reports of ethical misconduct, prompting the government to enact regulatory changes in response to ethical concerns and public activism. Some irresponsible conduct of research was related to conflicts of interest compromising ethics committee members’ objectivity and the inadequate training of lay members [26]. Despite the extensive documentation on the procedural aspects of informed consent, the study of the informed consent process in India remains significantly lacking [16]. Future research could focus on improving clarity checks in foreign languages, in this case, Indian langue in the documentation of essential documents in clinical trials [27–28].

In addition to the insufficient improvement of informed consent, the reporting of ethical considerations in publications is also characterized by underreporting. Cohen et al. (2009) arrived at the conclusion that pivotal ethical issues in clinical trials, particularly those conducted in developing countries, are inadequately disclosed in publicly accessible trial registries [35]. The crucial aspects of informed consent, justifiable placebo use, and post-trial obligations, which are vital for comprehending the trial conduct, remain undisclosed, leaving readers unaware of their existence. The absence of information regarding post-trial provisions for participant well-being and the increased likelihood of adverse events in LMICs further highlight the failure to address participants’ safety and benefit. However, the ethical debates surrounding standards of care and post-trial duties are complex and contentious, posing a challenging predicament for trialists and ethicists alike. Notably, the significant disparity in standards of care between developing and affluent nations, combined with the lack of specific guidelines, adds to the ongoing disagreement regarding the obligatory nature of ethical commitments.

### Moving forward

The clinical trial environment in LMICs may be improved by addressing a number of ethical issues and implementation techniques. First, integration with current health systems, public justification, and information framing is required, particularly for maternal immunization programme, which directly aligns with the United Nations Sustainable Development Goal (SDG) 3 (Good Health and Well-Being). To better understand the trial settings and increase participant comprehension throughout the informed consent process, formative, community-engaged research must be conducted at the start of new trials. Such participatory approaches not only enhance trust but also advance SDG 10 (Reduced Inequalities) by centering the rights of vulnerable populations, such as indigenous communities and pregnant women, in research design. It is crucial to verify the validity of informed consent procedures and assure the protection of trial participants [29]. In LMICs, better governance and culturally appropriate informed consent procedure modifications are also required. In another word, ethical harms in LMICs trials could be arise from actions of actors (for example, sponsors, investigators, deficient institutional processes) and from structural power imbalances between high-income and LMIC partners. Concepts such as “ethics dumping” and neo-colonial research practices are increasingly used to describe these phenomena [36]. This necessitates multidisciplinary collaboration between ethicists, policymakers, and social scientists to harmonize global ethical standards with local cultural norms—a priority underscored in global health equity frameworks.

Innovative solutions, such as digital consent platforms in local languages or AI-driven audits of ethical compliance, could address systemic gaps in transparency and participant understanding, aligning with the journal’s focus on health technology and informatics. Researchers must be culturally sensitive, mindful of legal ramifications, and take the community’s effect into account while working with indigenous populations [30]. Engaging indigenous leaders in co-designing trials ensures that research benefits extend equitably to these communities, reflecting the journal’s emphasis on social determinants and community health. It is also necessary to update the laws and rules governing ethical conduct in clinical studies involving indigenous communities. Policy reforms should prioritize institutionalizing ethical review frameworks within national health systems, ensuring accountability while respecting cultural autonomy, a critical step toward achieving SDG 16 (Peace, Justice, and Strong Institutions).

Future research should explore decentralized trial models and gender-responsive ethical guidelines to address barriers faced by women in LMICs, further advancing SDG 5 (Gender Equality). By embedding these strategies, LMICs can transition from being mere trial sites to equitable beneficiaries of research outcomes, fostering a paradigm shift toward justice-driven global health innovation.

### Strength and Weakness

The limitations of this overview include the potential author bias in the screening process, possibly leading to the neglect of some relevant articles and affecting the reliability and credibility of the overview. Additionally, the lack of systematic reviews in this overview may introduce a negative bias, and the absence of grey literature representation could result in publication bias and limited perspectives. Furthermore, the restriction to English language publications may limit the collection of ideas. For future projects, using relevant search terms such as ‘bioethics’ and ‘health research ethics’ should be considered. Comparing ethical concerns in clinical trials across different income countries, including low-income, lower- and upper-middle-income, and high-income countries, can provide insights to strengthen participant protection in LMICs. Furthermore, exploring ethical considerations in non-traditional clinical trials like digital or decentralized trials can be an important area for future literature to address the evolving landscape of clinical research in the digital era.

## 5 Conclusion

This umbrella review summarized and evaluated systematic-review evidence about ethical challenges in clinical trials in low- and middle-income countries. Across eight included reviews we identified three cross-cutting themes: (1) concerns for special and vulnerable populations (e.g., pregnant women, neonates, indigenous communities), (2) persistent problems with informed consent (including disclosure, comprehension, voluntariness and therapeutic misconception), and (3) deficits in research governance and underreporting of ethics procedures. Although informed consent was the most frequently reported issue, the methodological quality of the underlying reviews varied (AMSTAR-2 ratings ranged from high to critically low), and this heterogeneity tempers confidence in some findings.

Given these results, our principal recommendations are pragmatic and evidence-informed: strengthen REC capacity through competency-based training and administrative support; require standardized reporting of consent procedures and ethics oversight in trial publications; prioritize empirical research on neglected areas such as post-trial access and compensation for harm; and embed formative, community-engaged work into trial design to improve comprehension and trust. These steps, coupled with transparent monitoring of implementation, will better protect participants and help ensure that clinical research in LMICs contributes equitably to health gains.

## Supplementary Material

Supplementary Files

This is a list of supplementary files associated with this preprint. Click to download.

- SupplementaryfileTable.docx

## Figures and Tables

**Table 1:** Eight systematic reviews which were included in this study

Author	Year	Title
Beeler et al.^17^	2016	A systematic review of ethical issues in vaccine studies involving pregnant women
Raciti et al.^18^	2021	Ethical considerations for research involving pregnant women living with HIV and their young children: a systematic review of the empiric literature and discussion
De Pretto-Lazarova et al.^19^	2020	Informed consent approaches for clinical trial participation of infants with minor parents in sub-Saharan Africa: A systematic review
Patterson et al.^20^	2021	Informed consent rates for neonatal randomized controlled trials in low- and lower middle-income versus high-income countries: A systematic review
Fitzpatrick et al.^21^	2016	Seeking consent for research with indigenous communities: a systematic review
Tam et al.^22^	2015	Participants’ understanding of informed consent in clinical trials over three decades: systematic review and meta-analysis
Paramasivan et al.^23^	2021	What empirical research has been undertaken on the ethics of clinical research in India? A systematic scoping review and narrative synthesis
Cohen et al.^24^	2009	Reporting of informed consent, standard of care and post-trial obligations in global randomized intervention trials: a systematic survey of registered trials

**Table 2:** The characteristics of target populations and the associated ethical implications of the selected systematic reviews

Author	Population	Ethical implications
Beeler et al.^17^	Pregnant women	IRB approval
		Informed consent
		Disclosure of information
		Decision-making factor
		Cultural factor
		Study design/ scientific validity
Raciti et al.^18^	Pregnant women living with HIV, children	Informed consent
		Paternal involvement
		Accessibility to treatment
		Risk management
De Pretto-Lazarova et al.^19^	Children, minor parents	Informed consent
Patterson et al.^20^	Neonatal	Informed consent
Fitzpatrick et al.^21^	Indigenous communities	Informed consent
Tam et al.^22^	Participants in clinical trials	Informed consent
Paramasivan et al.^23^	Lay participants, professionals	Informed consent
		Knowledge of clinical trials, research ethics, and ethics committees
Cohen et al.^24^	Global randomized trials	Informed consent
		Standard of care
		Post-trial support
